# The impact of transfluthrin on the spatial repellency of the primary malaria mosquito vectors in Vietnam: *Anopheles dirus* and *Anopheles minimus*

**DOI:** 10.1186/s12936-019-3092-4

**Published:** 2020-01-06

**Authors:** Nicholas J. Martin, Vu S. Nam, Andrew A. Lover, Tran V. Phong, Tran C. Tu, Ian H. Mendenhall

**Affiliations:** 1 Naval Medical Research Unit TWO, Singapore, Singapore; 2 National Institute of Hygiene and Entomology, Ministry of Health, Hanoi, Vietnam; 3 Department of Biostatistics and Epidemiology, School of Public Health and Health Sciences, University of Massachusetts, Amherst, MA USA; 4 Duke-NUS Medical School, Programme in Emerging Infectious Diseases, 8 College Road, Singapore, 169857 Singapore

**Keywords:** Spatial repellent, Vietnam, *Anopheles dirus*, *Anopheles minimus*, Transfluthrin, Southeast Asia, Malaria elimination

## Abstract

**Background:**

The complexity of mosquito-borne diseases poses a major challenge to global health efforts to mitigate their impact on people residing in sub-tropical and tropical regions, to travellers and deployed military personnel. To supplement drug- and vaccine-based disease control programmes, other strategies are urgently needed, including the direct control of disease vectors. Modern vector control research generally focuses on identifying novel active ingredients and/or innovative methods to reduce human-mosquito interactions. These efforts include the evaluation of spatial repellents, which are compounds capable of altering mosquito feeding behaviour without direct contact with the chemical source.

**Methods:**

This project examined the impact of airborne transfluthrin from impregnated textile materials on two important malaria vectors, *Anopheles dirus* and *Anopheles minimus*. Repellency was measured by movement within taxis cages within a semi-field environment at the National Institute of Hygiene and Epidemiology in Hanoi, Vietnam. Knockdown and mortality were measured in adult mosquito bioassay cages. Metered-volume air samples were collected at a sub-set of points in the mosquito exposure trial.

**Results:**

Significant differences in knockdown/mortality were observed along a gradient from the exposure source with higher rates of knockdown/mortality at 2 m and 4 m when compared with the furthest distance (16 m). Knockdown/mortality was also greater at floor level and 1.5 m when compared to 3 m above the floor. Repellency was not significantly different except when comparing 2 m and 16 m taxis cages. Importantly, the two species reacted differently to transfluthrin, with *An. minimus* being more susceptible to knockdown and mortality. The measured concentrations of airborne transfluthrin ranged from below the limit of detection to 1.32 ng/L, however there were a limited number of evaluable samples complicating interpretation of these results.

**Conclusions:**

This study, measuring repellency, knockdown and mortality in two malaria vectors in Vietnam demonstrates that both species are sensitive to airborne transfluthrin. The differences in magnitude of response between the two species requires further study before use in large-scale vector control programmes to delineate how spatial repellency would impact the development of insecticide resistance and the disruption of biting behaviour.

## Background

Mosquito-borne infections are one of the largest burdens on human public health infrastructure and economic development, with more than half of the global population currently at risk for one or more infections [1]. This is especially relevant in Southeast Asia where multiple mosquito-transmitted diseases are endemic, including Chikungunya virus, dengue virus, Japanese encephalitis virus, malaria, and lymphatic filariasis [2]. Malaria remains an important contributor to morbidity and mortality, with an estimated 219 million cases and 435,000 deaths globally in 2017 [3]. While the majority of these cases are in Africa (90%), Southeast Asia contributes 3.5% of global cases [3] and is the only region with confirmed resistance in *Plasmodium falciparum* to artemisinin-based drug combination therapy [4]. Despite the presence of drug resistant malaria, many countries in Southeast Asia have made important strides towards their malaria elimination goals due to effective case detection and follow up, development of new drug combinations, and mosquito control interventions that disrupt the malaria transmission cycle [5]. The effectiveness of mosquito control interventions, like insecticide-treated bed nets (ITNs), have had limited effectiveness, due to several factors, including outdoor biting vectors and shifting vector behaviour. Addressing limitations in current mosquito control interventions is one of the critical factors for continued and accelerated progress [6].

Malaria cases in Vietnam have decreased substantially over the past three decades. In 1991, there were 1,672,000 clinical cases and 4650 deaths, while in 2015 this had been reduced to 19,252 clinical cases with 3 deaths [7]. In 2016, there were 4161 cases with approximately equal numbers of *P. falciparum* and *Plasmodium vivax* [8]. Both species are endemic throughout Vietnam. Historically *P. falciparum* caused the majority of disease burden in Vietnam, however similar numbers of *P. vivax* and *P. falciparum* cases have been reported in recent years [8, 9]. The majority of transmission events occur in the central highlands and along international borders where there has been substantial deforestation [9–11]. The primary mosquito vectors in Vietnam are *Anopheles dirus* and *Anopheles minimus*; both of these vectors are species complexes. *Anopheles dirus* is a long-lived species that is generally very anthropophilic, both important traits for vectors. These mosquitoes reside in forests, near mountainous regions, and prefer to oviposit in temporary, shaded pools [12]. *Anopheles minimus* is also a species complex, primarily found in the forested, hilly areas in Southeast Asia [12].

Vietnam has a goal to eliminate malaria by 2030 [13]. Malaria reduction involves targeting both the parasite and vector concurrently. The principal measures for malaria control and elimination include identifying and rapidly treating cases, performing indoor residual spraying (IRS), routine distribution of insecticide-treated nets (ITNs) and ensuring access to the most effective drug regimens. However, there are many challenges, including rapidly changing malaria transmission patterns and growing resistance to existing anti-malarial drugs. As the case load in Vietnam has fallen, it has become increasingly more difficult to detect and respond to foci [14]. This is especially true where infections are asymptomatic or below the limit of detection by routine microscopy. Additionally, the Greater Mekong Subregion is the epicentre of resistance to artemisinin and increasingly partner drugs [15, 16]. This is compounded by the availability of suboptimal therapies (including chloroquine) and counterfeit drugs [17]. For treatment of *P. vivax,* primaquine is effective to kill quiescent liver stages, but endemic areas of Vietnam have high prevalence of glucose-6-phosphate dehydrogenase deficiencies, with the potential for drug-induced toxicity [18].

Control of *Anopheles* vectors is an integral part of malaria control and requires integrated mosquito management (IMM), which leverages surveillance, source reduction, larval control, adult control, and resistance monitoring [19]. This is difficult, time-consuming and expensive, especially when there is mesoendemic transmission [20]. As larval habitats are difficult to locate, ephemeral, and scattered across the landscape, larvicides are not an efficient approach to vector control in this setting. This often leads to the reliance on ITNs and IRS to prevent contact between the vector and human or to kill the vector. These approaches can be effective, but can also lead to changes in mosquito resting (endophilic to exophilic) and biting (endophagic to exophagic) behaviours [21]. Additionally, the use of ITNs and IRS may lead to increased insecticide resistance with continued exposures to sub-lethal doses [22, 23]. With the absence of new insecticide classes, different approaches to modify vector behaviour are needed to prevent contact between vectors and humans. Spatial repellents are semi-volatile chemical compounds emitted from impregnated fabric or released by burning repellent-containing materials that elicit an excito-repellency effect in mosquitoes without direct contact with the treated material [24].

Spatial repellents’ mode of action is through odour receptors in the mosquito antennae and results in behavioural responses instead of the toxic effects observed during direct contact with repellent and insecticide compounds [25]. Thus, mosquitoes do not have to land on treated materials to be affected by the chemical compound and the continual release of these chemicals creates a barrier space, or buffer, between humans and vectors, preventing bloodmeal acquisition and consequent parasite transmission. To characterize the efficacy of spatial repellents on local vector species, a semi-field enclosure was created at the National Institute of Hygiene and Epidemiology (NIHE) in Hanoi. Repellency and knockdown/mortality of transfluthrin on colonies of both *An. dirus* and *An. minimus* was observed during trails with both species caged side-by-side. The effects of distance, height and differences in sensitivity between the two mosquito species are presented in this report.

## Methods

### Mosquito rearing and insecticide resistance testing

*Anopheles dirus* and *An. minimus* were reared at Vietnam National Institute of Malariology, Parasitology and Entomology and the National Institute of Hygiene and Epidemiology. These colonies have been maintained on-site from historical field collections. Eggs from colony-reared mosquitoes were hatched in deoxygenated water and larvae were fed a mixture of shrimp powder, bread powder, green bean powder, and vitamins. Adults were maintained on a 10% glucose solution, and females were blood fed 2–3 times per week through artificial membranes. Colonies were tested for susceptibility to pyrethroids every 6 months with insecticide-impregnated paper provided by the World Health Organization [26].

### Semi-field enclosure setup

A semi-field enclosure was established at NIHE. The enclosure was approximately 28 m long, 3 m wide and 3 m high. Burlap (hessian) fabric was impregnated with transfluthrin using a previously described method [27]. Briefly, transfluthrin was placed into a mixing container at a ratio of 10 mL of transfluthrin, 90 mL of dish washing detergent and 400 mL of water (2% treatment). Burlap fabric was soaked in the transfluthrin solution for 30 min, then hung inside the semi-field enclosure to dry. Two different sized impregnated fabric sheets were prepared and measured 3 m × 2 m or 3 m × 1 m. During trials the fabric was placed in the semi-field enclosure at one end of the enclosure. Ambient temperature and relative humidity were captured at 1-min intervals during all semi-field studies using a HOBO data logger (Onset Computer Corporation, Bourne, MA).

Taxis cages similar to those described previously, were built to measure mosquito movement up and down the anticipated spatial repellency (SR) concentration gradient [28]. Knockdown cages were built to restrict mosquito movement and measure toxic effects following exposure to airborne transfluthrin. After the fabric was placed in the enclosure mosquitoes were loaded into mosquito bioassay cages and taxis cages in the insectary. These cages were placed 2, 4, 8, 12, and 16 m away from the impregnated fabric. At each distance, a set of bioassay cages were placed at ground level, 1.5 m and 3 m to determine if there was a differential vertical distribution of the chemical. Each set of bioassay cages had 25 *An. dirus* and 25 *An. minimus,* while 50 specimens of each species was placed into each taxis cage. The taxis cages were then opened to allow for mosquito movement (Additional file 1: Figure S1). All taxis cages were cleaned with acetone between trials to minimize residual impacts on mosquitoes. Before the placement of the treated fabric, taxis boxes and five bioassay cages were placed at each distance in the semi-field enclosure to test for the presence of residual insecticides. No mortality was recorded. Three additional bioassay cages for each species were used to assess handling mortality and were kept in the insectary for monitoring. They were provisioned with water and a 10% sucrose solution.

### Collection of air samples

Air samples were collected during a subset of the mosquito exposure studies. These were taken at each height and distance of the bioassay cages. Samples were collected using pre-conditioned stainless-steel tubes (89 mm × 4 mm i.d. × 6.4 mm o.d.) packed with 100 mg of Tenax-TA adsorbent and 100 mg of Carbograph 5TD adsorbent (CAMSCO; Houston Tx, USA). Personal air sampling pumps (Gilair 5000; Syensidyne; St Petersberg, Fl, USA) were set to operate with a flow rate of 1000 mL/min for 60 min. Pumps were calibrated to within 5% of set flow rate (1000 mL/min) prior to sampling using a device to measure volumetric flow rate (Defender 510, Bios International, Butler, NJ). Air samples were collected for the duration of mosquito exposure to the treated fabric, brass caps with inert liners were placed on the tubes at the conclusion of sampling. Tubes were stored at 2–8 °C prior to sample analysis. Samples were analysed by a commercial analytical laboratory (Maxxam Analytics, Novi, Michigan) using EPA Method TO-17.

### Collection of mosquitoes and monitoring of knockdown and mortality

Mosquitoes were exposed to the fabric for 60 min, after which the taxis cages were shuttered to prevent additional movement and then removed from the semi-field enclosure. Bioassay cages were brought back to the insectary where all mosquitoes were transferred to holding containers and mosquito knockdown and mortality was recorded at 0 h, 1 h, 4 h, 8 h, and 24 h post-exposure. The total number of mosquitoes not moving was recorded for knockdown (mosquitoes immobile on the floor of the cage); and at 24-h post-exposure, the total number of dead mosquitoes was recorded. Taxis cages were placed in direct sunlight to kill the mosquitoes after which they were collected and identified to determine if they were in the attracted, neutral or repellent section of the taxis cage. Bioassay cages were disposed of and empty taxis cages were washed with acetone to remove residual insecticide products. The fabric was kept in the semi-field enclosure until the next trial. The exposure trial with mosquitoes was repeated on days 1, 3, 5, 7, and 9 after treatment of the fabric.

### Statistical analysis

Analysis of single proportions (mortality or knockdown) used fractional response models with robust standard errors to account for clustering of treatments; the ‘fracreg’ package was used [29, 30]. Model fit was assessed used both the Akaike/Bayesian information criteria (AIC/BIC), and McFadden’s adjusted R^2^ was used to assess the exploratory power of models [31]. To quantify the impacts of transfluthrin on vector taxis, the total vectors activated (that is either attracted or repelled over the total in each replicate) was first calculated and this proportion compared between treatment conditions [32]. Secondly, this ‘total activated’ then used as the denominator for the proportion attracted (with the corresponding ‘repelled’ showing identical but reversed effect sizes). All analyses utilized Stata 15.1 (College Station, TX); and all tests are two-tailed with α = 0.05.

## Results

### Mortality and knock-down

**Fig. 1:**
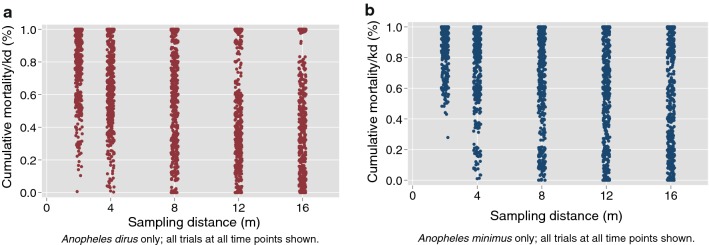
Relationship between proportion mortality/knock-down and sampling distance. Mean values across all time points, pooled across replicates (**a** *An. dirus* and **b** *An. minimus*)

**Fig. 2:**
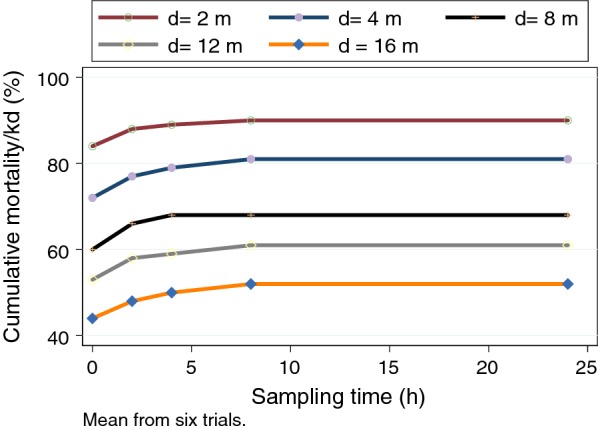
Relationship between proportion mortality/knock-down and sampling time at each distance, by vector species, pooled across replicates

**Fig. 3:**
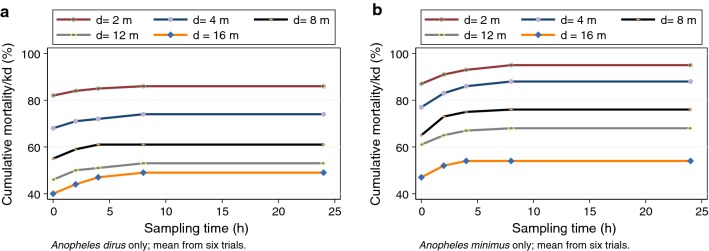
Proportion of vectors repelled, attracted or with neutral movement from transfluthrin source at each experimental sampling distance, by species (**a** *An. dirus* and **b** *An. minimus*)

The proportion of vectors showing either mortality or knock-down (hereafter combined as mortality/kd) at each sampling distance is shown for both vector species (Fig. 1). These plots suggest an inflection point near 2–4 m, and important differences in response to transfluthrin between the vector species. The corresponding plots by sampling time can be found in Additional file 1: Figure S1, but show limited variability. Qualitatively, comparisons of the unadjusted (no covariates) relationship between distance and proportion with mortality/knock-down by species (Fig. 2). Examining both species together, the maximum mortality/knock-down was observed within 2-h at the 2-m and 4-m distances, with longer times (4 h) at the 8-m and 16-m distances. Species-specific survival curves at each sampling distance demonstrate that at sampling points closest to the transfluthrin source, mortality/knock-down reaches high levels rapidly, but was attenuated at the 12 m and 16 m distances (Fig. 3).

A set of multivariable regression models was used to fully quantify the relationships between sampling distance, sampling height, vector species and other experimental factors, on the proportion of vectors exhibiting mortality/kd. For a few replicates, the totals with mortality/knock-down decreased between time points; whether this was due to measurement error, or recovery from knock-down is unknown. There was no mortality in the controls), leading to an inability to estimate risk in the treatment; as such it was not possible to estimate the impact of treatment directly. At all distances, a distinct plateau of effects can be observed near 2–4 h from the start of exposure. This suggests that the airspace has saturated and maximal mortality/knock-down has occurred. Proportionate mortality was lower in *An. dirus* at all distances in comparison with *An. minimus*, and the initial slopes of the curves (exposure-time relationships) appear qualitatively different between the two species at all distances in Additional file 2: Figure S2, Additional file 3: Figure S3, Additional file 4: Figure S4.

These models suggest that when adjusted for distance and sampling height, mortality/knock-down drops off extremely rapidly after the first few time periods: relative to the 2-h sampling time, there is a 33-fold greater mortality/knock-down at time zero, and then the remaining periods have lower mortality/knock-down of approximately 40% and one-third at the 4-h and 8-h time points. The relationship of sampling distance with mortality/kd, with adjustment for covariates, shows at the 2-m sampling has 3.4-fold greater mortality/knock-down relative to 16-m, with a clear increase at each sampling distance. The sampling height results also suggest a drop-off away from the floor- using the 2 m sampling point as the reference, the 1.5 m sample shows a 50% higher risk of mortality-kd, which increases to about double at floor height (Table 1).

**Table 1:** Multivariable fractional response general linear models for interval-based proportion of vectors with mortality/knock-down (errors adjusted for experimental replicates)

Factor	Relative proportion ratio	95% CI	*p* value
Sampling time (h)			
0	32.69	27.11–30.42	< 0.001
2	Ref.	–	–
4	0.42	0.35–0.50	< 0.001
8	0.32	0.26–0.39	< 0.001
24	2.9 e−0.8	2.49 e−08–3.25 e−08	< 0.001
Sampling distance (m)			
2	3.41	2.91–4.00	< 0.001
4	3.25	2.37–4.46	< 0.001
8	2.10	1.57–2.81	< 0.001
12	1.26	0.91–1.75	0.161
16	Ref	–	–
Sampling height (m)			
0	1.93	1.69–2.20	< 0.001
1.5	1.53	1.33–1.76	< 0.001
2.0	Ref	–	–
Species			
*Anopheles dirus*	Ref	–	–
*An. minimus*	1.31	1.18–1.45	< 0.001
Square type			
1 × 3	Ref	–	–
2 × 3	2.08	1.69–2.56	< 0.001
Percent RH (median daily)			
Numeric	0.996	0.99–1.00	0.071
Temperature, F (median daily)			
Numeric	1.01	1.001–1.021	0.025

There are important quantitative differences between the response of the two different vector species to transfluthrin, with *An. minimus* showing 1.31-fold higher risk of mortality/knock-down relative to *An. dirus* when adjusted for covariates. The use of the 2 × 3 square type for insecticide release was associated with a 2.1-fold higher risk of mortality/knock-down relative to the 1x3 square type. These models were also adjusted for the median daily temperature and relative humidity; the RH was not associated with mortality/kd; and an increase of the median daily temperature of one degree Fahrenheit was associated with a 1% increase in mortality. Finally, these models explain a high proportion of the variability in these data (pseudo R^2^ = 0.48), suggesting that this set of covariates captures many important facets of these experiments.

Analogous multivariable models were also used to quantify the impact of measured transfluthrin levels for the trial where air sampling was undertaken (Table 2). The truncation of transfluthrin levels at 20 ng does not allow for use of quantitative transfluthrin levels in the multivariable models. As such, the values below the limit of detection of 20 ng were compared with all stations having measurable transfluthrin values (Additional file 5: Figure S5 and Additional file 6: Figure S6). These models suggest that when adjusted for distance and sampling height, mortality/knock-down drops off extremely rapidly after the first few time periods: relative to the 2-h sampling time, there is a 49-fold greater mortality/knock-down at time zero, and then the remaining periods have lower mortality/knock-down of approximately one-third at the 4-h and 8-h time samples, and very low at 24-h.

**Table 2:** Multivariable fractional response general linear models for interval-based proportion of vectors with mortality/knock-down with inclusion of transfluthrin sampling data (errors adjusted for experimental replicates)

Factor	Relative proportion ratio	95% CI	p-value
Sampling time (h)			
0	48.53	32.39–72.73	< 0.001
2	Ref.	–	–
4	0.37	0.23–0.60	< 0.001
8	0.36	0.22–0.60	< 0.001
24	1.33 e−0.7	9.48 e−08–1.87 e−07	< 0.001
Sampling distance (m)			
2	5.26	3.31–8.35	< 0.001
4	4.59	1.46–14.44	0.009
8	2.44	0.81–7.35	0.114
12	1.78	0.63–5.03	0.275
16	Ref	–	–
Sampling height (m)			
0	2.25	1.62–3.12	< 0.001
1.5	1.83	1.33–2.51	< 0.001
2.0	Ref	–	–
Species			
*Anopheles dirus*	Ref	–	–
*An. minimus*	1.48	1.13–1.92	0.003
Transfluthrin detected			
No	Ref		
Yes (≥ 20 ng)	1.57	1.23–2.01	< 0.001
Percent RH (median daily)			
Numeric	0.997	0.80–1.01	0.747
Temperature, F (median daily)			
Numeric	0.90	0.84–0.97	0.007

The relationship of sampling distance with mortality/kd, with adjustment for covariates, shows 5.3-fold greater mortality/knock-down at 2-m compared to 16-m, with an increase at each closer distance. The sampling height results also suggest a drop-off away from the floor, using the 2 m sampling point as the reference, the 1.5 m sample shows 1.8-fold higher risk of mortality-kd, which increases to 2.3-fold at floor-level. Adjusted for covariates, there are important quantitative differences in the response in the vectors to transfluthrin, with *An. minimus* showing 1.5-fold higher risk of mortality/knock-down relative to *An. dirus*. Most importantly, this set of replicates allows adjustment for measured transfluthrin levels from concurrent air sampling, and stations with measurable levels (≥ 20 ng) showed a 1.6-fold greater mortality/knock-down per time period relative to those below 20 ng when adjusted for sampling distance and environmental covariates. Finally, these models explain a high proportion of the variability in these data (pseudo R^2^ = 0.46), suggesting that this set of covariates captures much of the variation in these experiments, despite some limitations in measured transfluthrin levels.

### Mosquito taxis studies

**Fig. 4:**
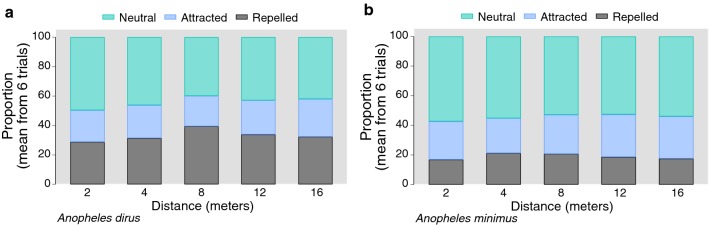
Proportion of total vectors showing mortality or knock-down by sampling distance; all time points and heights shown. (N = 25 mosquitoes per replicate) (**a** *An. dirus* and **b** *An. minimus*)

To quantify the impact of distance from the transfluthrin source on observed taxis behaviour, plots showing the proportion of vectors moving in each of three categories at different distances, averaged across all trials (Fig. 4). These plots show that at essentially all of the sampled distances, approximately half of mosquitoes showed no measurable response and remained in the ‘neutral’ movement category with clear differences in response between the two different vector species. Some variation was also observed between trial replicates (Additional file 7: Figure S7, Additional file 8: Figure S8, Additional file 9: Figure S9).

**Fig. 5:**
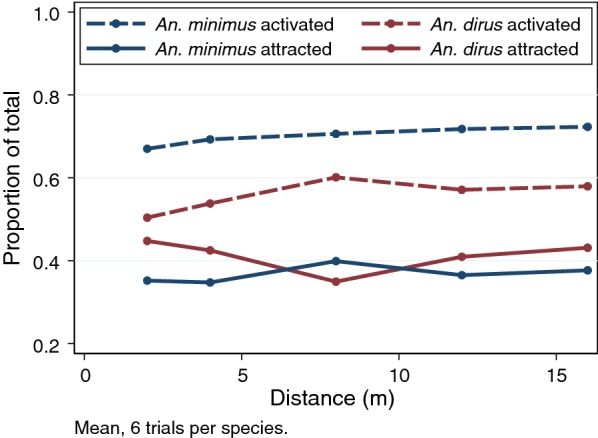
Comparison of the proportion of total vectors that showed any activation (either repelled or attracted); and proportion of those that were attracted out of the total that were activated (see “Methods”)

To qualitatively assess the proportion of vectors that were attracted or repelled from the origin, the proportion of all vectors that were activated (showing any taxis) were compared with the proportion of those with any taxis that were repelled is shown, with mean values from the replicates (Fig. 5). The proportion activated was consistent across distances for both species, (~ 50–60% for *An. dirus*, and ~ 70% for *An. minimus*) when averaged across trials. Comparison of the proportion showing any taxis for each trial can be found in the Additional file 5: Figure S5 (both species combined) and Additional file 6: Figure S6; Additional file 7: Figure S7 (individual plots), and suggest large variation between the trials, which should be considered for future work.

Multivariable models were used to assess the proportion activated [(total repelled + total attracted)/(total vectors)] (Table 3). These models suggest that there is a steep drop-off in risk of activation by increasing sampling time and that sampling distance has no impact (except for 2-m vs. 16-m, with p = 0.031). There is a large and significant important difference in the risk of activation between the two vectors, with *An*. *minimus* showing 1.91-fold greater risk of activation relative to *An. dirus* when adjusting for covariates. This may be due to increased taxis observed in *An. minimus* during these trials or due to increased mortality/knockdown observed in *An. dirus.*

**Table 3:** Multivariable fractional response general linear models for proportion of total vectors activated (showing either repellency from or attraction to source) (errors adjusted for experimental replicates)

Factor	Relative proportion ratio	95% CI	p-value
Time (h)			
0	Ref.	–	–
2	0.065	0.040–0.11	< 0.001
4	0.0067	0.037–0.012	< 0.001
8	0.0056	0.0032–0.010	< 0.001
24	1.6 e−0.9	1.19e−09–2.13e−09	< 0.001
Distance (m)			
2	0.74	0.58–0.98	0.031
4	0.85	0.69–1.05	0.131
8	1.01	0.89–1.15	0.869
12	0.98	0.83–1.13	0.680
16	Ref	–	–
Species			
*Anopheles dirus*	Ref	–	–
*An. minimus*	1.91	1.51–2.41	< 0.001
Trial			
1	Ref	–	–
2	2.36	2.32–2.40	< 0.001
3	3.50	3.43–3.57	< 0.001
4	2.10	2.07–2.12	< 0.001
5	1.70	1.69–1.72	< 0.001
6	1.38	1.37–1.38	< 0.001

These models have not accounted for any concurrent mortality/knock-down at each timepoint, and lack of impact by sampling distance may be due to saturation of responses in the anophelines. Models for proportion activated with inclusion of measured transfluthrin values (above and below the limit of detection) had unchanged conclusions, and the transfluthrin variable was not significant (p = 0.15).

Models were used to assess predictors for the proportion repelled out of all vectors showing taxis [(total repelled)/(total repelled + total attracted)] (Table 4). The risk of repellency did not show any association with increasing distance aside from 8-m vs 16-m, which may be a statistical artifact (p = 0.011). There was a statistically significant difference between the species with *An. minimus* showing 1.21-fold higher risk of repellency relative to *An. dirus*. However, these models showed very limited exploratory power (adjusted-R^2^ = 0.01), suggesting limited ability to capture biologically important variation with these covariates. Recent modelling studies of taxis experiments using *Aedes aegypti* suggest highly complex and non-linear responses to SRs in these vectors [33], which may partially explain the low exploratory power of these models.

**Table 4:** Multivariable fractional response general linear models for proportion of vectors showing repellency from the total of all vectors exhibiting taxis (errors adjusted for replicates and trial)

Factor	Relative proportion ratio	95% CI	p-value
Distance (m)			
2	1.02	0.85–1.21	0.846
4	1.08	0.95–1.21	0.217
8	1.13	1.03–1.25	0.011
12	1.07	0.94–1.23	0.299
16	Ref	–	–
Species			
*Anopheles dirus*	Ref	–	–
*An. minimus*	1.21	1.13–1.30	< 0.001

### Air sampling results

Ninety air samples were collected proximal to mortality and taxis cages during a subset of mosquito response trials. The mean sampling time was 62.26 min (SD = 3.71 min) and the mean sample volume collected was 62.24 L (SD = 3.70 L). Tubes where one or both brass endcaps were disturbed during shipment to the analytical laboratory were not included in final analysis. Transfluthrin air concentrations ranged from non-detectable to 1.32 ng/L. The majority of evaluable air samples (83.3%) were below the method limit of quantitation for the method (20 ng). The spatial arrangement sampling air sampling values is shown in Additional file 8: Figure S8 and Additional file 9: Figure S9.

## Discussion

Spatial repellency has increasingly been recognized as an important intervention to impact the transmission of mosquito-borne diseases [34] and the World Health Organization has provided guidelines on how to accurately assess if a product will be effective [35]. These compounds are typically botanical extracts or synthetic pyrethroids (including transfluthrin, allethrin or metofluthrin) [34, 36, 37] selected due to their combination of volatility and relatively low toxicity to mammals [38]. Multiple studies evaluating the impact of spatial repellents on mosquito behaviour and mortality have been conducted on various species of mosquitoes, including; *Aedes aegypti*, *Aedes albopictus, Aedes canadensis, Aedes vexans, Culex quinquefasciatus* and several species of *Anopheles* mosquitoes (*Anopheles gambiae* and *An. minimus*) [27, 39–43]. However, these studies have focused on a single mosquito species, were conducted in the field with unknown mosquito populations, or compared multiple mosquito species tested in separate experiments. This report includes the side-by-side comparison of two anopheline species under controlled conditions allowing for direct comparison of mortality and repellency between the species. This is critical to understanding the effectiveness a spatial repellent in areas with multiple vector species that may have different sensitivity to toxic (measured by mortality/kd) and repellent products [34].

Mosquitoes encountering spatial repellents often exhibit excito-repellency as well as acute and chronic toxic consequences that can help ameliorate vector-borne transmission. Mosquitoes encountering these chemicals can be knocked down or can be killed, depending on the exposure and chemical [44]. In this study, there was significant mortality in the mosquitoes in the exposure cages, suggesting airborne transfluthrin induced toxic effects and not excito-repellency. However, either effect (excito-repellency or toxic) can disrupt mosquito biting and reduce the amount of blood that mosquitoes imbibe, potentially increase the time for host-seeking, and reduce attempts to blood feed, whether they are topical repellents (DEET) or those used as spatial repellents [32, 45, 46].

The specific impact of spatial repellent on exposed mosquitoes may be less important for disease control than the disruption of biting behaviour. Additional impacts of exposure to sub-lethal concentrations of spatial repellents may impact the fecundity of mosquitoes exposed to these products [47]. These downstream impacts were not assessed in this study, yet may also mitigate the overall impact of vector borne disease transmission through sublethal impacts.

Responses to spatial repellents have been shown to vary by mosquito species; permethrin, but not deltamethrin, was shown to have a repellent effect on *An. minimus*, yet neither had a measurable impact on *Aedes aegypti* [43]. These variable responses to different chemical compounds require that that each one should be validated against specific species before implementation in field trials as chemicals that act strongly on certain species, may show limited, or no impacts, on others [48, 49]. Additionally, toxicity is more likely to occur at higher concentrations of airborne chemical, often in small confined spaces or in very close proximity to the treated material, resulting in greater contact with the airborne chemical and immediate knockdown or mortality [44]. At low exposure levels, vectors may still enter dwellings with spatial repellents, but can be repelled or suffer knockdown [50, 51]. Exposure to sublethal doses can select for resistance with partially or fully resistant mosquitoes surviving exposure and becoming the dominant mosquito strain in areas of continued insecticide use [52, 53].

It is probable that there were heterogeneous concentrations of transfluthrin in the test space resulting in varied exposure conditions at the various heights and distances during tests in this study. Understanding the impact of varied concentrations of airborne chemical on inducing mosquito repellency, knockdown and/or death is critical for vector control programme planning. There is a concern that incomplete coverage with spatial repellents could result in mixed protection, suggesting a need for complete or near-complete coverage in a village because mosquitoes may be pushed into adjacent domiciles that lack impregnated fabric [36]. However, recent field studies suggest this may be of limited importance for volatilized transfluthrin-based approaches [54].

To determine if and how these chemicals can be used as spatial repellents, there are several factors that must be examined during evaluation. The chemicals must be analysed for their impact on the target mosquito species and what is the optimal concentration of airborne chemical to achieve repellency. Being able to characterize the concentration of active ingredient is important and during this trial, the collection tubes more often than not had concentrations below the method limit of detection (< 20 ng) and corresponding concentration (0.32 ng/L). The inability to analyse all air samples and the high proportion of evaluable samples with results below the limit of detection suggest the air sampling method used for this study was not sensitive enough to detect the transfluthrin in the air. However, the limited air sampling data can be used to define a potential air transfluthrin concentration range associated with mosquito toxicity.

The evaluation of exposure to spatial repellent compounds to multiple mosquito species is needed to inform vector control policy. In this study, two important malaria vector species were exposed to airborne transfluthrin that was generated from treated textile materials, a method potentially scalable for public health programmes. Transfluthrin-based spatial repellents are being developed for use to control vector populations that are poorly targeted by current vector control tools (“residual transmission”) [55, 56]. Knockdown and mortality were observed in both species, with important differences noted, suggesting that any transfluthrin-based intervention would impact both malaria vectors. Repellency, measured by significant numbers of mosquitoes moving away from the exposure source, was not consistently observed across the species or exposure heights and distances during these trials. This suggests that exposure to the concentrations of transfluthrin generated in this semi-field system resulted in knockdown and mortality and not repellency. All effects have the potential to disrupt biting behaviour and with consequent health impacts; however, optimizing spatial repellent intervention to repel, not kill, mosquitoes may help to reduce the total chemical burden in homes, and odour receptor medicated repellency may not drive resistance to the chemical. Future studies should implement these methods to assess the performance in field settings to demonstrate real world efficacy, target air transfluthrin concentrations below 0.32 ng/L using a more sensitive analytical method and/or larger sample volumes, and continue to include multiple disease vector species to better quantify the impact of varied exposure conditions on relevant disease vectors.

## Conclusion

The results from this study demonstrate that members of both of the primary malaria vector complexes in Vietnam are sensitive to transfluthrin-impregnated fabric, with some important differences in susceptibility. Future studies should examine efficacy and subsequent effectiveness of these tools work in field settings. As Vietnam moves to eliminate malaria by 2030, it is critical to identify additional tools, such as spatial repellents, that can accelerate progress especially in areas of residual transmission where the impact of current tools may be ineffective.

## Supplementary information

**Additional file 1: Figure S1.** Bioassay cage and air sample collection tube (A), taxis cage (B), and experimental setup (C).

**Additional file 2: Figure S2.** Proportion of total vectors showing mortality or knock-down at each sampling time; all distances and heights shown. (N= 25 mosquitoes per replicate) (A: *An. dirus* and B: *An. minimus*).

**Additional file 3: Figure S3.** Relationship between proportion mortality/knock-down and sampling distance for each sampling time. Mean values for both species combined, and all heights.

**Additional file 4: Figure S4.** Relationship between proportion mortality/knock-down and sampling distance for each sampling time, by species (A: *An. dirus* and B: *An. minimus*).

**Additional file 5: Figure S5.** Sampling tubes at and below transfluthrin LOD, trial day 2/5 (Trial 3).

**Additional file 6: Figure S6.** Sampling tubes at and below transfluthrin LOD, trial day 4/5 (Trial 3).

**Additional file 7: Figure S7.** Proportion of all vectors being activated (A) or repelled (B), both species combined, across trial replicates (showing median value, and interquartile range).

**Additional file 8: Figure S8.** Proportion of *An. dirus* vectors being activated (A) or repelled (B) across trial replicates (showing median value, and interquartile range).

**Additional file 9: Figure S9.** Proportion of *An. minimus* vectors being activated (A) or repelled (B) across trial replicates (showing median value, and interquartile range).

## Data Availability

The datasets used and/or analysed during the current study are available from the corresponding author on reasonable request.

## Abbreviations

DEET: *N*,*N*-diethyl-meta-toluamide
IRS: indoor residual spraying
ITS: insecticide-treated nets
kd: knock down
L: litre
ng: nanogram
NIHE: National Institute for Hygiene and Epidemiology
RH: relative humidity
SD: standard deviation
SR: spatial repellency
