# Supplementary figures and images for: The impact of transfluthrin on the spatial repellency of the primary malaria mosquito vectors in Vietnam: Anopheles dirus and Anopheles minimus
Source: Malar J. 2020 Jan 6;19:9. doi: 10.1186/s12936-019-3092-4 (PMC6945573; doi:10.1186/s12936-019-3092-4)

A

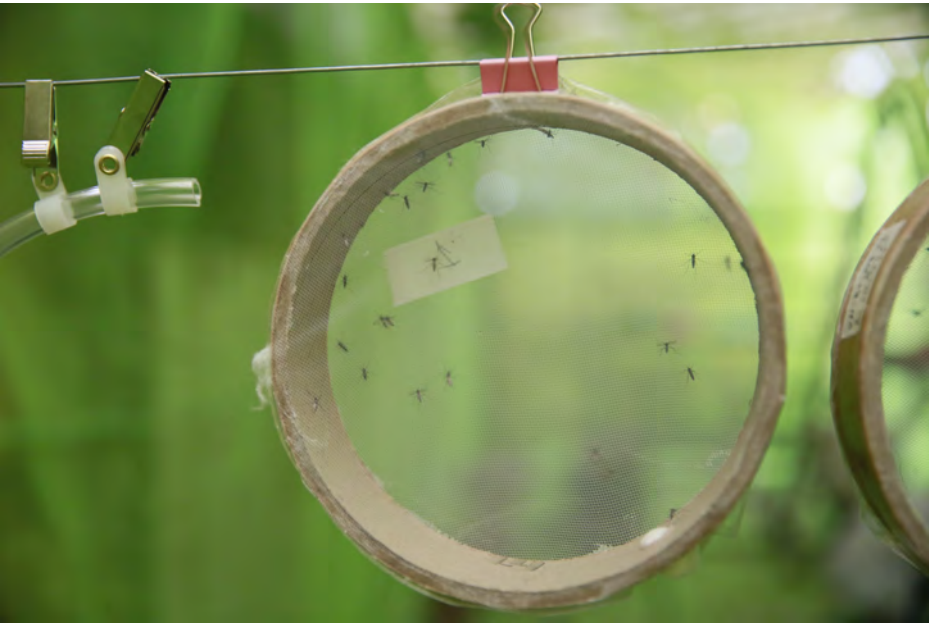

B

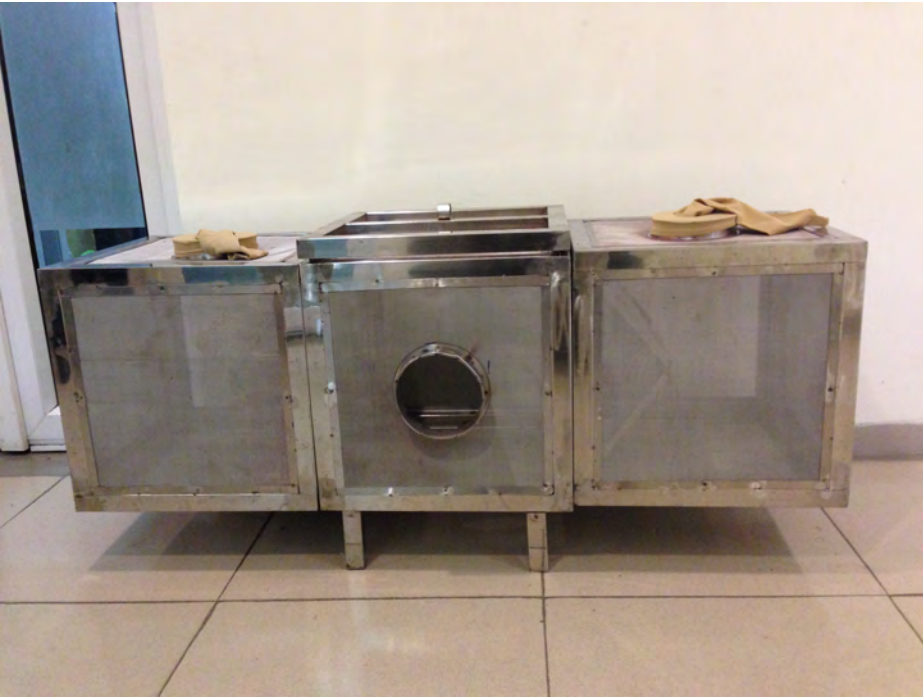

C

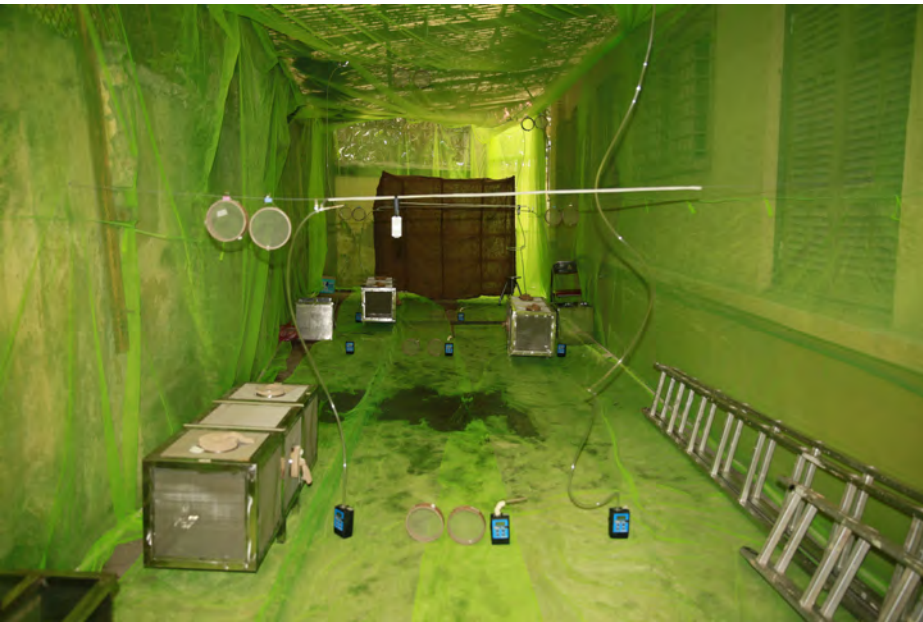

Supplement: Supplementary file 1 — Additional file 1: Figure S1. Bioassay cage and air sample collection tube (A), taxis cage (B), and experimental setup (C). [file 12936_2019_3092_MOESM1_ESM.pdf]

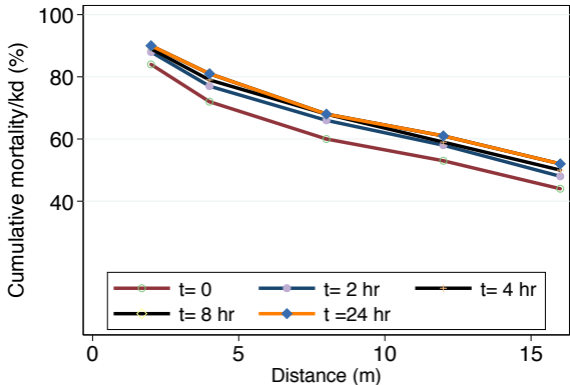

Mean from six trials.

Supplement: Supplementary file 3 — Additional file 3: Figure S3. Relationship between proportion mortality/knock-down and sampling distance for each sampling time. Mean values for both species combined, and all heights. [file 12936_2019_3092_MOESM3_ESM.pdf]

A

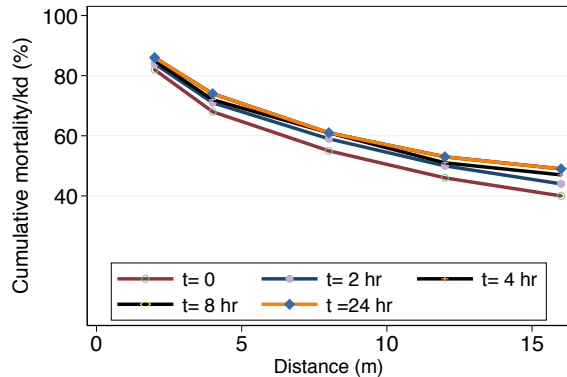

*Anopheles dirus* only; mean from six trials.

B

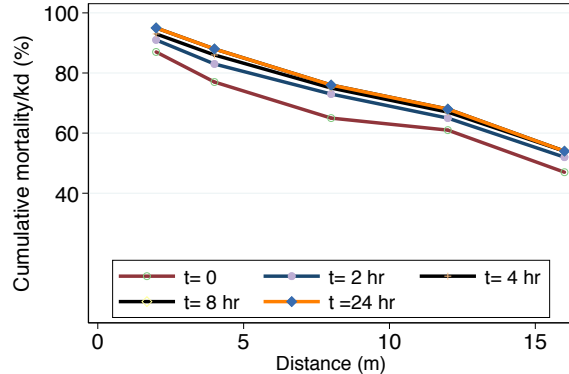

*Anopheles minimus* only; mean from six trials.

Supplement: Supplementary file 4 — Additional file 4: Figure S4. Relationship between proportion mortality/knock-down and sampling distance for each sampling time, by species (A: An. dirus and B: An. minimus). [file 12936_2019_3092_MOESM4_ESM.pdf]

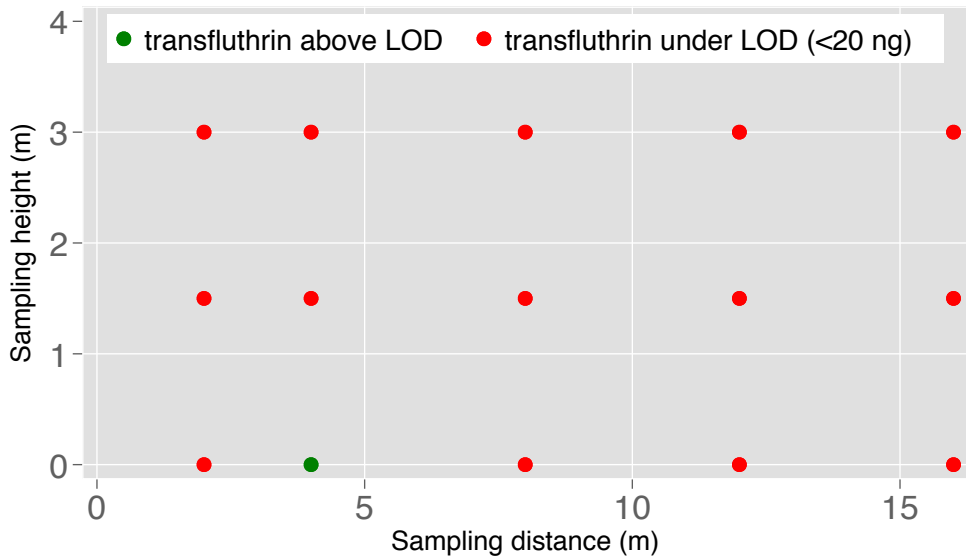

Data collected Oct. 27, 2017.

Supplement: Supplementary file 5 — Additional file 5: Figure S5. Sampling tubes at and below transfluthrin LOD, trial day 2/5 (Trial 3). [file 12936_2019_3092_MOESM5_ESM.pdf]

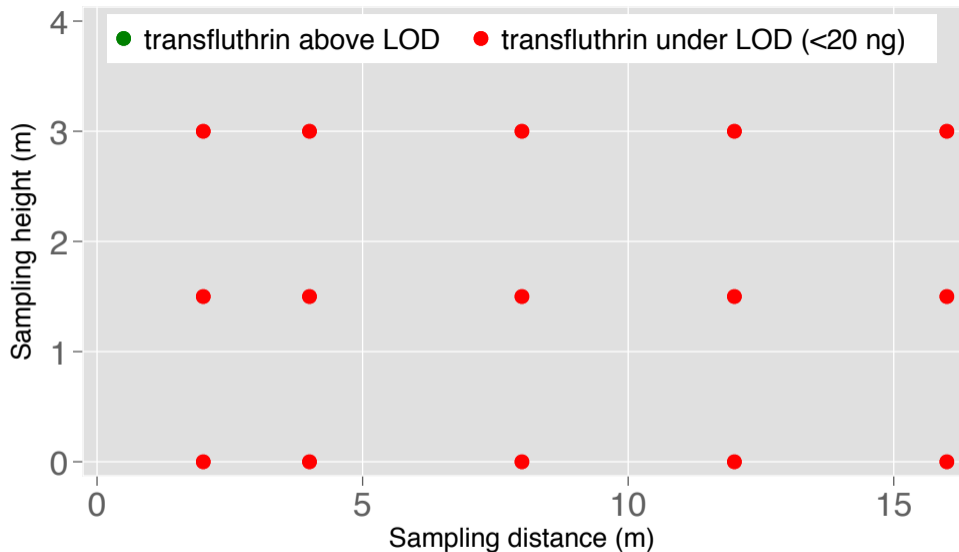

Data collected Nov. 1, 2017.

Supplement: Supplementary file 6 — Additional file 6: Figure S6. Sampling tubes at and below transfluthrin LOD, trial day 4/5 (Trial 3). [file 12936_2019_3092_MOESM6_ESM.pdf]

A

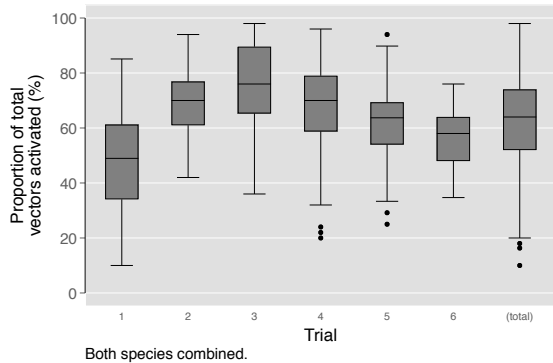

B

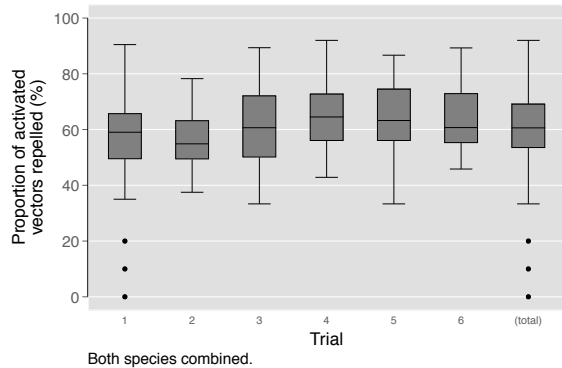

Supplement: Supplementary file 7 — Additional file 7: Figure S7. Proportion of all vectors being activated (A) or repelled (B), both species combined, across trial replicates (showing median value, and interquartile range). [file 12936_2019_3092_MOESM7_ESM.pdf]

A

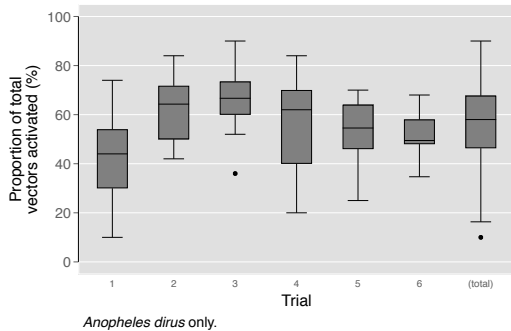

B

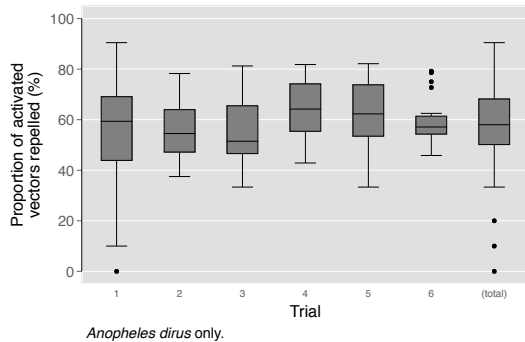

Supplement: Supplementary file 8 — Additional file 8: Figure S8. Proportion of An. dirus vectors being activated (A) or repelled (B) across trial replicates (showing median value, and interquartile range). [file 12936_2019_3092_MOESM8_ESM.pdf]

A

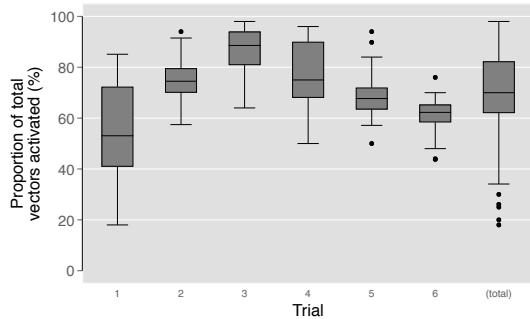

*Anopheles minimus* only.

B

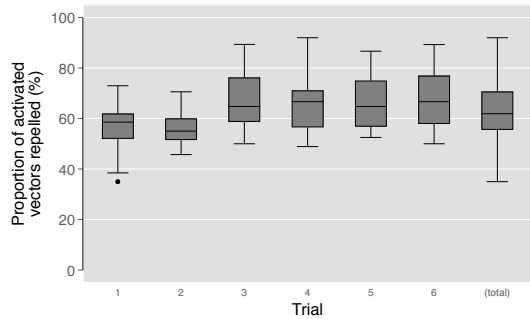

*Anopheles minimus* only.

Supplement: Supplementary file 9 — Additional file 9: Figure S9. Proportion of An. minimus vectors being activated (A) or repelled (B) across trial replicates (showing median value, and interquartile range). [file 12936_2019_3092_MOESM9_ESM.pdf]
