# Supplementary material for: The impact of transfluthrin on the spatial repellency of the primary malaria mosquito vectors in Vietnam: Anopheles dirus and Anopheles minimus
Source: Malar J. 2020 Jan 6;19:9. doi: 10.1186/s12936-019-3092-4 (PMC6945573; doi:10.1186/s12936-019-3092-4)
Supplement: Supplementary file 2 — Additional file 2: Figure S2. Proportion of total vectors showing mortality or knock-down at each sampling time; all distances and heights shown. (N= 25 mosquitoes per replicate) (A: An. dirus and B: An. minimus). [file 12936_2019_3092_MOESM2_ESM.pdf]

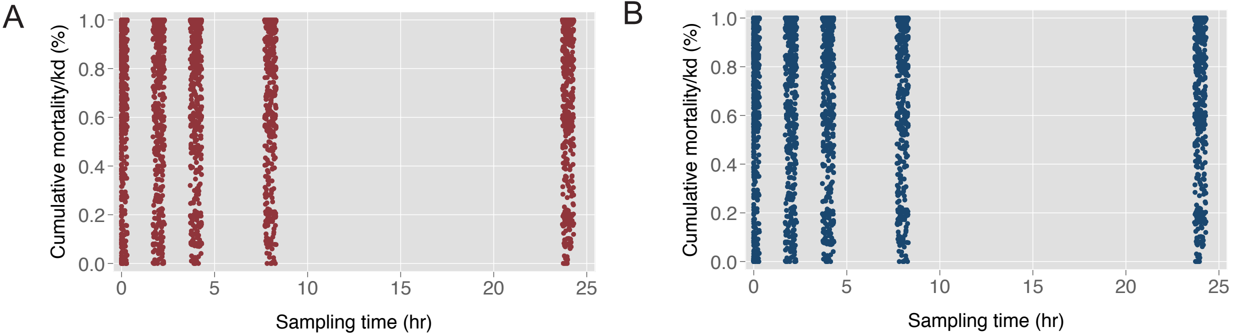

(All trials; all distances shown; *Anopheles dirus* only)

(All trials; all distances shown; *Anopheles minimus* only)
